# Possible reluctance to shorten antibiotic duration in Gram-negative bacteremia and limitations of mortality-based outcomes: the need to prioritize clinical-microbiologic recurrence in future trials—Insights from the “Bacteremia Antibiotic Length Actually Needed for Clinical Effectiveness” (BALANCE) Trial

**DOI:** 10.1016/j.ijregi.2025.100639

**Published:** 2025-03-20

**Authors:** Samadhi Patamatamkul

**Affiliations:** Department of Medicine, Suddhavej Hospital, Faculty of Medicine, Mahasarakham University, Maha Sarakham, Thailand

**Keywords:** Shorter durations, Bacteremia, Suppurative complications

## Abstract

Shorter antibiotic durations (≤7 days) have demonstrated non-inferiority to longer courses for several bacterial infections, but evidence for bacteremia remains limited. Trials often exclude patients with bacteremia, focus on uncomplicated cases, or lack sufficient power to detect clinically significant effects. The recent Bacteremia Antibiotic Length Actually Needed for Clinical Effectiveness (BALANCE) trial, a multicenter study spanning 74 hospitals, investigated 7 versus 14 days of antibiotic therapy for bloodstream infections, showing non-inferiority in 90-day all-cause mortality. Despite these findings, a possible reluctance to adopt shorter durations persists, as seen in high exclusion rates and protocol deviations. BALANCE highlights the importance of source control in managing bloodstream infections resulting in the relatively low 7-day mortality. However, reliance on 90-day mortality may underestimate clinical failure, with outcomes like suppurative or distant complications and recurrence being more relevant. The trial predominantly included non-severely immunocompromised patients with community-acquired Gram-negative bacteremia, limiting generalizability to multidrug-resistant or hospital-acquired infections. The BALANCE trial, along with the previous three randomized control trials comparing short- versus longer-duration antibiotics for Gram-negative bacteremia, supports guideline recommendations for shorter antibiotic courses in cases involving non-multidrug-resistant organisms, non-severely immunocompromised patients, and effective source control. It also highlights the importance of future trials prioritizing clinically meaningful outcomes and underrepresented populations.

## Highlights

• Shorter antibiotic durations are effective for most Gram-negative bacteremia.
• Physician reluctance and protocol deviations impact adherence to shorter courses.
• Mortality-based outcomes may underestimate clinical failure in bacteremia trials.

Shorter durations of antibiotic treatment (≤7 days) have been shown to be non-inferior to longer durations for several bacterial infections, including pneumonia, uncomplicated intra-abdominal infections (IAIs), pyelonephritis, and cellulitis [[1], [2], [3], [4], [5]]. However, evidence supporting shorter antibiotic courses for bacteremia remains limited, as many trials have excluded patients with bacteremia, focused on uncomplicated cases, or were modest in size, making it difficult to detect or exclude clinically significant effects [[6], [7], [8], [9]]. However, the most recent trial, “Antibiotic Treatment for 7 versus 14 Days in Patients with Bloodstream Infections (BALANCE),” conducted by Daneman et al. [10] and published in the *New England Journal of Medicine* on November 20, 2024, represents a significant achievement in shedding light on the standard recommendation for shorter durations of antibiotic treatment in bacteremia. Conducting a large, multicenter trial spanning 74 hospital sites across seven countries is a commendable accomplishment. Moreover, the authors diligently addressed the gap by recruiting over 55% critically ill or intensive care unit patients, a population underrepresented in the previous three randomized control trials (RCTs) on antibiotic duration for Gram-negative bacteremia [[7], [8], [9], [10]] (Table 1). The investigators deserve recognition for addressing the longstanding and complex question of optimal antibiotic duration for bloodstream infections, a subject often clouded by entrenched myths advocating prolonged courses.

**Table 1:** Summary of major RCTs comparing shorter versus longer durations of antibiotics for Gram-negative bacteremia.

	Yahav *et al.* [7]	von Dach *et al*. [8]	Molina *et al*. [9]	Daneman *et al.* [10]
**Target population**	Uncomplicated Gram-negative bacteremia	Uncomplicated Gram-negative bacteremia	Uncomplicated Gram-negative bacteremia	Bloodstream infection
**Design**	Open-label/analyst-blinded multicenter RCT	Open-label multicenter RCT	Open-label multicenter RCT	Open-label multicenter RCT
**Screened population**	4,807	NA	1,496	36,637
**Assessed for eligibility, n (%)**	2,169 (45.1%)	2,345	NA	13,597 (37.1%)
**Random, n (%)**	604 (27.8%)	504 (21.5%)	248 (16.6%)	3,631 (26.7%)
**Had attending physician decline to participate, n (%)**	150/2,169 (6.9%)	42 (1.8%)	None	4,532/13,597 (33.3%)
**Excluded due to uncontrolled source, n (%)**	912/4,807 (19%)	NA	327/1,496 (21.9%)	NA
**Non-inferiority margin**	10%	10%	10% (for clinical outcome)	4%
**Intensive care unit or critically ill patients, %**	NA	NA	NA	55%
**Median SOFA score**	2	1 (qSOFA)	NA	4
**Charlson comorbidity score, median**	2	1	45.4% ≥3	4 (clinical frailty scale score)
**Immunosuppression**	22.5%^a^	excluded	18.6%^b^	12.7%^c^
**Source of bacteremia**				
Urinary	69.3%	63%	59.3%	41.7%
Intraabdominal or hepatobiliary	12.1%	22%	13.6%	18.6%
Respiratory	4.6%	8%	2.5%	12.6%
**Hospital-acquired infection**	26.5%	27%	30.5%	23.9%
**Source control procedure**	NA	NA	25.9%	43.8%
**Microorganism**				
*Escherichia coli*	60.8%	74%	66.4%	44.4%
*Klebsiella pneumoniae* or *Klebsiella* spp.	15.3%	17%	17.6%	15.0%
*Enterobacter* spp.	NA	2%	9.2%	6.6%
Multidrug-resistant organism	18.9%	6.5% (ESBL)	17% (ESBL and AmpC)	NA
**Primary outcome**	Composite outcome of 90-day all-cause mortality and clinical failure	Clinical failure by day 30	Total number of days of antibiotics treatment	90-day all-cause mortality
**Mortality outcome**				
30-day mortality	**5.2% vs 4%**	**3.7% vs 2.4%**	**3.4% vs 6.3%**	NA
90-day all-cause mortality	**11.8% vs 10.7%** risk difference 1 (−4.0 to 6.1)	**8.9% vs 5.6%**	**8.5% vs 11.8%**	**14.5% vs 16.1%** risk difference −1.6 (−4.0 to 0.8)
**Clinical or microbiologic outcome**				
Relapse of bacteremia	2.6% vs 2.7%	9% vs 22%	6.5% vs 5%	2.6% vs 2.2%
Distant complications	0.7% vs 0.3%	0% vs 0%	NA	NA
Absence of clinical cure	NA	6.6% vs 5.5%	7.3% vs 9.8%	NA
Suppurative complications	5.2% vs 3.4%	18% vs 11%	NA	NA
**Resistance development**	**10.8% vs 9.7%** risk difference 1.0 (−3.7 to 5.9)	NA	NA	**9.5% vs 8.5%** risk difference 1.1 (−0.8 to 2.9)^e^
**Adverse events**				
Diarrhea	5.9% vs 8.1%	NA	1.7% vs 2.3%	NA
Acute kidney injury	4.6% vs 4%	0% vs 0.6%^d^	2.5% vs 0.8%	0.8% vs 0.9%
*Clostridioides difficile* infection	1% vs 0.3%	1.2% vs 2.4%	NA	1.7% vs 2%

a Prednisolone ≥20 mg/day or equivalent, solid organ transplant, or stem cell transplant.

b Solid organ transplant and immunosuppressant drugs.

c Chemotherapy or prednisolone ≥15 mg/day or equivalent.

d Elevated creatinine.

e Secondary infection or colonization with antibiotic-resistant organisms. ESBL, extended-spectrum β-lactamase; NA, not available; RCT, randomized control trial; SOFA, Sequential Organ Failure Assessment. Percentages in the table are derived from the baseline characteristics of the short-duration arm. The sequence of percentage comparisons is short-duration arm versus long-duration arm.

Nevertheless, a noteworthy observation from BALANCE is the possible reluctance of physicians to adopt shorter antibiotic courses, in particular Gram-negative bacteremia. Previously, in a trial conducted by Yahav et al. [7] looking at the outcome of shorter antibiotics duration for uncomplicated Gram-negative bacteremia, a total of 150 out of 2,169 (6.9%) eligible participants were excluded due to treating physicians’ declining participation. This reluctance was even more pronounced in BALANCE, where 33% (4,532 of 13,597) of eligible patients were excluded for the same reason [10]. Similarly, a survey revealed substantial variation in treatment durations among infectious disease specialists, with 15% favoring longer durations (≥10 days), particularly for pneumonia, vascular infections, and IAIs [11]. These contrast with other non-Gram-negative trials, with or without bacteremia, where the reasons for physician denial to participate were clearly absent [1,3,5]. This highlights possible skepticism among clinicians, despite growing evidence supporting shorter durations, particularly for uncomplicated Gram-negative bacteremia [12,13]. The trial's substantial protocol deviations further underscore this hesitation. The difference between the intention-to-treat (ITT) and per-protocol (PP) populations (a total of 755 patients across both arms, 20.9%, with 23.1% of patients in the shorter-duration arm receiving a longer duration than 7 days, with an overall median of 8 days (interquartile range 8-11 days) reflects significant deviations, such as patients receiving antibiotics longer than the assigned duration [10]. One of the reasons for the high exclusion rate by treating physicians may be the lack of data on shorter antibiotic durations in critically ill patients, a gap that the BALANCE trial has finally addressed [10].

Examining the primary outcome of death from any cause within 90 days by ITT, 261/1,802 (14.5%) in the 7-day group versus 286/1,779 (16.1%) in the 14-day group, with a difference of −1.6% (95% confidence interval −4.0 to 0.8), is promising. Although ITT analysis is the gold standard for non-inferiority trials, such deviations likely biased the results towards the “non-inferiority” hypothesis. Conversely, the PP analysis, despite reduced power and loss of randomization, demonstrated no increased mortality in the 7-day group, further supporting the trial's findings [10]. However, these deviations in the ITT analysis may have diminished the ability to detect true non-inferiority, although the PP analysis provides supportive evidence but cannot replace the primary ITT analysis.

To further explore the likely cause of non-inferiority in this trial and similar studies, it is essential to consider the fundamental principles of infectious diseases. Source control is a critical component in managing any infections including bloodstream infections, particularly in cases such as obstructive uropathy causing urinary tract infections (UTIs), skin and soft tissue infections requiring drainage, or IAIs, including obstructive hepatobiliary diseases necessitating intervention. However, BALANCE does not provide clarity on what constituted source control (e.g., percutaneous nephrostomy for obstructive uropathy, incision and drainage for skin and soft tissue infection, or endoscopic retrograde cholangiopancreatography for cholangitis) or analyze outcomes based on source control status within infection types. Addressing these gaps could enhance understanding of how source control interacts with antibiotic duration to influence outcomes.

This raises the question of whether 90-day all-cause mortality, as the primary outcome, is truly appropriate to capture the real difference between short and long durations of antibiotic therapy. Although it is a robust outcome measure for most sepsis-related studies and an important objective outcome in non-placebo-controlled studies, its relevance in this context may be less pronounced. For bloodstream infections managed with early comprehensive care, effective source control, and appropriate antibiotics, the acute infection's impact on 90-day mortality is likely minimal. This is supported by supplementary data from BALANCE, which reported impressively low 7-day mortality of 0.8% in a population with a median Sequential Organ Failure Assessment score of 4 [10], far below the 10% observed in other studies of patients with sepsis with similar scores [14,15]. Some experts argue that the BALANCE trial, by allowing patient randomization up until day 7, inherently excludes many early fatalities. This approach is an expected departure from trials that focus on early interventions. Although this is partially true, it underscores the necessity for the trial to have explicitly reported which individuals died before recruitment [10]. Without precise data from the trial, both hypotheses remain plausible: whether the impressively low 7-day mortality rate resulted from perfect source control, or whether the most severe cases had already succumbed before recruitment.

Consequently, reliance on 90-day all-cause mortality may underestimate the clinical failure of shorter durations. Exploratory outcomes such as suppurative complications (e.g., renal abscess in pyelonephritis or empyema in pneumonia), distant complications (e.g., growth of the same pathogen in new locations), and organ-based recurrence (e.g., relapse UTI or post-cholangitis abscess requiring re-intervention) might better capture the clinical trade-offs between short and long antibiotic courses. This is evident in previous trials by Yahav et al*.* [7] and von Dach et al*.* [8], which demonstrated trends toward higher rates of suppurative complications (5.2% vs 3.4%; and 1.2% vs 0.6%) and distant complications (0.7% vs 0.3%; and 0% vs 0%) in the short-duration group compared with the long-duration group, respectively. The previous trial by Molina et al. also demonstrated a higher incidence of relapsing fever from a worsening of the infection compared with the control in the PP analysis (6.5% vs 4.7%) [9]. However, these differences were deemed insignificant due to the lack of statistical power in the studies [16]. One might argue that these clinical outcomes may seem insignificant and less feasible to conduct due to the need for extensive follow-up and the associated cost burden. In the context of demonstrating clinical failures of short-duration antibiotics, it is crucial to pursue this research, given the significant costs of complications and rehospitalizations and their impact on the quality of life of patients [17,18]. In addition, most RCTs, including the BALANCE trial [[7], [8], [9], [10]], involve Gram-negative bacteremia originating from a urinary source, where commonly used antibiotics achieve high urinary concentrations (Table 1). Because bacteremia itself does not indicate a poorer prognosis for UTIs, using mortality as the outcome may not accurately reflect true clinical failure. An ongoing BALANCE trial substudy is investigating this issue, which will provide valuable insights and help close this gap [19]. An alternative approach to clinical outcomes in infectious disease trials is the use of desirability of outcome ranking outcomes, with or without response-adjusted for antibiotic risk [20]. This was demonstrated in the trial by Molina et al. [9], where the authors used a composite outcome of clinical response, adverse events, and death, finding a higher probability of a better outcome in the shorter-duration groups. This approach is particularly relevant for future trials on antibiotic duration in bloodstream infections, where mortality alone may not be a feasible primary outcome.

Finally, the BALANCE trial primarily included non-severely immunocompromised patients with community-acquired bloodstream infections treated with third-generation cephalosporins, meropenem, piperacillin-tazobactam, or ciprofloxacin, representing 77% of cases [10]. This indicates that the majority of infections were caused by non-multidrug-resistant (non-MDR) organisms, aligned with most previous trials [[6], [7], [8], [9], [10],^12^]. This focus on non-MDR infections suggests that the findings may not be directly applicable to settings with a higher prevalence of MDR pathogens, particularly hospital-acquired infections. Notably, only 23.9-30.5% of nosocomial infections were included in the previous three RCTs on Gram-negative bacteremia and the BALANCE trial [10], with 6-18.9% classified as MDR organisms [[7], [8], [9]] (Table 1). Moreover, monomicrobial infections of Gram-negative origin accounted for 63.4%, whereas UTIs and IAI/hepatobiliary infections combined comprised 50.5% [10]. Therefore, caution is warranted when generalizing these findings to other populations. However, these data are also reassuring for physicians to adopt a shorter duration of antibiotics when all these conditions are met.

The BALANCE trial is a significant contribution, challenging entrenched beliefs and advancing the evidence base for shorter antibiotic courses. Given the current evidence, guidelines should further support the adoption of shorter antibiotic durations for most cases of community-acquired Gram-negative bacteremia originating from urinary or intra-abdominal sources, provided early source control is feasible. In light of the increasing incidence of *Clostridiodes difficile* infection, antibiotic-related adverse events such as acute kidney injury and diarrhea, and the potential for resistance development, a shorter duration is considered safe and effective from an antimicrobial stewardship perspective [[7], [8], [9], [10],^13^]. Although no clear significant development of resistant organisms was observed in the BALANCE trial or the study by Yahav et al., it is crucial to note that these trials did not incorporate active surveillance to detect such outcomes, and many cases of resistance development may have been missed [7,10] (Table 1). Future studies addressing these considerations, including the use of clinical outcomes that better reflect the clinical failure of short antibiotic durations and a greater focus on Gram-positive infections and non-UTI/IAI sources, are eagerly anticipated.

## Declarations of competing interest

The author have no competing interests to declare.
